# Brain and blood transcriptome profiles delineate common genetic pathways across suicidal ideation and suicide

**DOI:** 10.1038/s41380-024-02420-z

**Published:** 2024-01-26

**Authors:** Shengnan Sun, Qingkun Liu, Zhaoyu Wang, Yung-yu Huang, M. Elizabeth Sublette, Andrew J. Dwork, Gorazd Rosoklija, Yongchao Ge, Hanga Galfalvy, J. John Mann, Fatemeh Haghighi

**Affiliations:** 1 Department of Neuroscience, Icahn School of Medicine at Mount Sinai, New York, NY 10029 USA; 2 James J. Peters VA Medical Center, Bronx, NY 10468 USA; 3 Department of Psychiatry, Columbia University, New York, NY 10032 USA; 4 Division of Molecular Imaging and Neuropathology, New York State Psychiatric Institute, New York, NY 10032 USA; 5 Department of Neurology, Icahn School of Medicine at Mount Sinai, New York, NY 10029 USA

**Keywords:** Molecular biology, Neuroscience, Genetics

## Abstract

Human genetic studies indicate that suicidal ideation and behavior are both heritable. Most studies have examined associations between aberrant gene expression and suicide behavior, but behavior risk is linked to the severity of suicidal ideation. Through a gene network approach, this study investigates how gene co-expression patterns are associated with suicidal ideation and severity using RNA-seq data in peripheral blood from 46 live participants with elevated suicidal ideation and 46 with no ideation. Associations with the presence of suicidal ideation were found within 18 co-expressed modules (*p* < 0.05), as well as in 3 co-expressed modules associated with suicidal ideation severity (*p* < 0.05, not explained by severity of depression). Suicidal ideation presence and severity-related gene modules with enrichment of genes involved in defense against microbial infection, inflammation, and adaptive immune response were identified and investigated using RNA-seq data from postmortem brain that revealed gene expression differences with moderate effect sizes in suicide decedents vs. non-suicides in white matter, but not gray matter. Findings support a role of brain and peripheral blood inflammation in suicide risk, showing that suicidal ideation presence and severity are associated with an inflammatory signature detectable in blood and brain, indicating a biological continuity between ideation and suicidal behavior that may underlie a common heritability.

## Introduction

Between 2010 and 2020, suicide was the 12th most common cause of death in the US [1]. Suicidal behaviors contribute to a significant health and economic burden, exceeding $70 billion per year in the United States alone (CDC, 2020). Although genetics, together with environmental and other individual factors, contribute to suicidal risk [2], family and twin studies have been foundational in establishing the genetic contribution to suicidality. Such studies show a higher frequency of suicide attempt and death in monozygotic twins compared to dizygotic twins among suicide twin survivors but not non-suicide twin survivors [3]. Family studies suggest a significant genetic contribution with heritability ranging from 30 to 55% for suicidal ideation (SI), behavior, and death [4, 5], with additive genetic effect on the continuum of the phenotype spanning suicidal ideation, behavior, and death [6].

Clinical studies link more severe SI, like that characterized by a plan and intent, to imminent risk of suicide attempts. Most studies have examined associations between aberrant gene expression and suicide behavior, but consideration of how such transcriptional profiles align with SI and evidence for a suicidal behavior-ideation phenotype continuum is lacking. Transcriptional studies that capture the spectrum of suicide risks using peripheral blood and brain samples can capture common elements across the molecular pathologies between ideation and suicidal behavior that may underlie a common phenotypic heritability [5, 6]. Such research may provide a genomic explanation for suicidal behavior as a risk factor for future suicidal acts [7]. To intervene effectively and efficiently, new methods are needed to identify not just who is at risk, but also identify, using dynamic markers, times when they are at highest risk. This remains challenging, as individual vulnerability to suicide appears to be complex and multidimensional, with many contributing sociodemographic, genetic, and environmental factors [8–10].

In recent years, there has been increasing interest in harnessing genomic technologies and large-scale genomic data to improve understanding of the underlying biology of suicide. One approach used to study the biology of suicide is to examine gene transcript expression patterns. Gene transcription is a functional output of genes expressed in a molecular pathway or process. Biological signals inducing gene transcript expression can vary in timescales from milliseconds to seconds, hours, days, or even decades with varying transcriptional kinetics [11]. Like the observed temporal dynamics in transcriptional activity, SI is also an inherently dynamic construct [12, 13]. As such, expression activity of genes associated with SI in comparison to expression patterns in the brains of suicide decedents can provide an important snapshot of the underlying biology contributing to the intensity of the suicidal state. Complex phenotypes of suicide may reflect interactions of multiple intertwined genes, transcription factors, and gene co-expression clusters (i.e. genes with correlated levels of expression) in cells with modular but diverse functional patterns [14]. Co-expression gene clusters represent coherent functional pathways in both normal conditions [15] as well as in disease states [14]. To capture such gene co‐expression clusters, gene network approaches have been widely applied in systems biology and brain research across psychiatric and neurodegenerative diseases as depression [16], bipolar disorder [17], schizophrenia [18], and Alzheimer’s disease [19, 20].

Although gene network approaches have been applied in transcriptome studies of suicide in human postmortem brain tissue from suicide decedents [21], to our knowledge, no study has investigated transcriptome profiles of SI in clinical samples using gene co-expression network methods to uncover biological processes and pathways underlying SI. As SI and gene transcripts are both highly dynamic, identification of gene networks associated with SI or its severity could be of utility in clinical translational studies for the development of blood biomarker profiles that can be used to identify individuals at risk for suicide. To this end, for the first aim of the present study, the Multiscale Embedded Gene Co-Expression Network Analysis (MEGENA) [22] method was used to identify co-expressed gene modules associated with SI and severity, endorsed within two weeks prior to blood collection. The second aim of the present study was to determine whether these SI-associated co-expressed modules were also differentially expressed in gray and white matter postmortem human brain specimens of suicide decedent cases and non-suicide controls, to determine which elements of peripheral blood gene expression changes related to SI were also found in brain gene expression related to suicide death. This common pathophysiology may reflect common heritable elements between ideation and suicide or perhaps the intense suicidal ideation at the time of suicide.

## Methods

### Samples & subjects

All studies were approved by the Institutional Review Board (IRB) of the New York State Psychiatric Institute, IRB number for live subjects is #4815 and postmortem studies is 7351 R.

### Live subject sample recruitment and evaluation

Study participants were adults 20–65 years old and were recruited in the New York metropolitan area by advertising, via the Columbia University Medical Center Portal for research subject volunteers and through patient referrals from clinics and mental health professionals. Participants provided written informed consent as approved by the IRB of the New York State Psychiatric Institute (see above). Participants were evaluated at the New York Psychiatric Institute. Study participants comprised of three groups: 1. Healthy controls (HC, *n* = 27); 2. participants with a major depressive disorder (MDD) diagnosis with no history of suicide attempt (MDD/NS, *n* = 50); and 3. participants with MDD diagnosis with history of suicide attempt (MDD/SA, *n* = 23) (Table 1). All participants were evaluated using the Structured Clinical Interview for DSM-IV (SCID I) [23] by trained clinicians, with raters being at least Master’s level psychologists. MDD participants included in the study met DSM-IV criteria for current major depressive episode in context of MDD based on the SCID, Axis I and II disorders were assessed using Structured Clinical Interviews for DSM-IV and Structured Clinical Interviews for DSM-IV Axis II Disorders [24]. Healthy volunteers included in the study had no personal history of Axis I disorders, cluster B personality disorders, substance use disorder and lifetime history of suicide attempt, and had no first-degree relatives with a history of mood disorder, psychotic disorder, or suicidal behavior. Participants were excluded if they presented with a history of psychosis, serious active medical illness, substance use disorder in the 6 months prior to enrollment, or pregnancy. Taking psychotropic medication was not an exclusion criterion for the depressed groups.

**Table 1:** Demographic and clinical characteristics for 100 subjects for MEGENA network building.

	Total N = 100	MDD/SA N = 23	MDD/NS N = 50	HC N = 27	P-value	Sig. pair
**Age**, Mean ± sd	36.4 ± 11.4	36.3 ± 11.1	37.5 ± 11.2	34.7 ± 12.3	0.5906	NA
**Sex**, n(%)						
Male	40 (40%)	8 (35%)	21 (42%)	11 (41%)	0.8393	NA
Female	60 (60%)	15 (65%)	29 (58%)	16 (59%)		
**Ethnicity**, n(%)						
Hispanic	17 (17%)	3 (13%)	8 (16%)	6 (22%)	0.7342^#^	NA
Non-Hispanic	83 (83%)	20 (87%)	42 (84%)	21 (78%)		
**Race**, n(%)						
White	59 (59%)	15 (65%)	31 (62%)	13 (48%)	0.2138^#^	NA
Black or African American	23 (23%)	5 (22%)	8 (16%)	10 (37%)		
Asian	11 (11%)	3 (13%)	7 (14%)	1 (4%)		
Other	7 (7%)	0 (0%)	4 (8%)	3 (11%)		
**Smoking Status**^**1**^						
Moderate	2 (2%)	1 (4%)	1 (2%)	0 (0%)	0.2047^#^	NA
Light	7 (7%)	3 (13%)	4 (8%)	0 (0%)		
Non-smoker	90 (90%)	19 (83%)	44 (88%)	27 (100%)		
**Age of Onset of Major Depression**		16.5 ± 8.9	21.5 ± 13.0		0.1419^#^	NA
**Number of episodes of Major Depression**	3.6 ± 5.8	4.9 ± 5.5	4.6 ± 6.4	0 ± 0	<0.0001^#^	MDD/NS > HC, MDD/SA > HC
**RNA Integrity Number (RIN)**	7.3 ± 1.0	7.3 ± 1.0	7.3 ± 1.0	7.5 ± 1.0	0.7750^#^	NA
**Beck Scale for Suicidal ideation (SSI),** Mean ± sd	6.7 ± 8.6	12.6 ± 9.0	7.6 ± 8.4	0 ± 0	<0.0001^#^	MDD/NS > HC, MDD/SA > HC
**Beck Depression Inventory (BDI),** Mean ± sd	17.1 ± 14.8	27.2 ± 12.0	22.1 ± 12.6	0.9 ± 2.2	<0.0001^#^	MDD/NS > HC, MDD/SA > HC
**Hamilton Depression Rating Scales (HAM17),** Mean ± sd	13.6 ± 10.2	20.9 ± 5.8	19.9 ± 5.2	1.3 ± 1.5	<0.0001^#^	MDD/NS > HC, MDD/SA > HC
**Suicidal ideation group**, n^2^						
SSI = 0 (No-SI)	46	4	15	27		
SSI ≥ 5 (High-SI)	46	19	27	0		
0 < SSI < 5 (Low-SI)	6	0	6	0		

Demographic and clinical characteristics were compared across the three groups using one-way ANOVA or Kruskal-Wallis test when appropriate for continuous measurements and when there significant was group difference, followed by Tukey HSD test or Wilcoxon rank sum test with Bonferroni correction correspondingly. Chi-squared test or Fisher’s exact test were performed for count measurements when appropriate. # non-parametric tests.

^1^Smoking status information was missing for one subject in MDD/NS group.

^2^Low-SI subjects and 2 subjects from MDD/NS group who did not have SSI total score were excluded for SI group comparison.

Depressive symptoms were assessed by a research clinician using the 17-item Hamilton Depression Rating Scale (HAM17) [25]. Patients’ subjective perception of depression severity was assessed by means of the Beck Depression Inventory (BDI) [26]. Suicide attempt was defined as a self-injurious act with some degree of intent to end one’s life. Severity of suicide attempts was characterized by using the Suicide Intent Scale [27], which assessed the patient’s expectation regarding the outcome of the suicidal behavior, and the Lethality Rating Scale [27], which assessed the degree of medical injury resulting from the attempt. Suicide attempts did not involve interrupted or aborted attempts. Psychiatric diagnoses and suicide attempts were verified in a consensus conference with research psychologists and psychiatrists. The number, method and degree of medical damage of past suicide attempts were recorded on the Columbia Suicide History Form [28]. Suicidal ideation was measured by the Beck Scale for Suicidal ideation (SSI) [29]. Moderate to elevated SI was defined as SSI ≥ 5 corresponding to reported suicidal ideation in the last two weeks prior to the assessment, and no ideation was defined by SSI = 0. All subjects underwent physical examinations, where data collected included such information as smoking history and degree, and history of past and current medications (Table S1). Blood was collected from all study participants using RNA Paxgene vacutainer tubes.

### Postmortem brain samples

Brain samples were obtained from the Macedonian/New York State Psychiatric Institute Brain Collection in the Molecular Imaging and Neuropathology at the New York State Psychiatric Institute. Cause of death, excluding suicide, was determined by the medical examiner. All cases and controls were psychiatrically characterized by psychological autopsies, using a validated psychological autopsy interview method with at least one significant other [30]. Diagnoses of major psychiatric disorders were determined using the SCID I [31] and suicide was determined using the Columbia Classification Algorithm for Suicide Assessment [32]. Suicide decedents did not involve interrupted or aborted attempts. Suicide decedents had a lifetime diagnosis of MDD, while sudden death controls did not have an Axis I psychiatric disorder. The postmortem human brain samples included specimens from gray and white matter tissues. Prefrontal cortical regions, specifically the dorsal gray matter (BA 9) and ventral white matter (BA 47), from frozen brain sections were used for RNA-seq assays. RNA-seq data from gray matter cases with 29 controls and 21 suicide decedents were downloaded from GEO database accession# GSE101521, with details on cause of death, postmortem interval (PMI), and brain toxicology described previously [33]. Briefly, gray matter cases died suddenly, were free of gross neuropathology and had negative brain toxicology for psychotropic, illicit psychoactive drugs, and neurotoxic drugs [33]. White matter cases consisted of 15 suicide decedents and 9 non-psychiatric non-suicide controls who died of accidental causes and were all free of gross neuropathology. Among the 15 white matter suicide cases, the cause of death included 10 (67%) Hanging, 3 (20%) intoxication, 1 (7%) gunshot, and 1 (7%) falling from height (Table S2). Among the 9 controls, The cause of death included 5 (56%) cardiomyopathy, 1 (11%) gunshot, 1 (11%) industrial accident, 1 (11%) stabbing, and 1(11%) vehicle accident (see supplemental Table S2 for details on brain toxicology, as well as subject demographics and PMI and brain pH).

### RNA sequencing

For live samples, PAXgene blood tubes were thawed quickly and brought to room temperature prior to total RNA isolation according to the manufacturer’s instructions, and Globin mRNA was removed using GLOBINclear (Invitrogen/Ambion). For postmortem human brain specimens, total RNA was isolated from ~30–50 mg white matter tissue using RNeasy Lipid Tissue Mini Kit (Qiagen, #74804) according to the manufacturer’s instructions. RNA quality was measured via BioAnalyzer and RNA integrity Number (RIN) for live and white matter postmortem samples is reported in Table 1 and Table S2, respectively. All RNA samples were subject to Ribo-zero depletion, and libraries were prepared using the Illumina Truseq library preparation kit and sequenced on the Illumina HiSeq 2500 (2 × 50), generating a mean of 48 million reads per sample. Reads were aligned to hg19 using STAR aligner and annotated to transcripts using Gencode v18 annotation.

### Statistical and network analysis

Demographic and sample-related characteristics were compared between the two groups for both gray and white matter samples using two-sided *t* test or Wilcoxon rank-sum test for continuous measurements and Chi-squared test or Fisher’s exact test for count measurements when appropriate and compared across three groups for live samples (see Table 1). Moreover, RNA-seq data for the live samples was filtered based on expression level and sample variability. Lowly-expressed genes with 0.5 count per million (CPM) in fewer than 2% of samples or with average log_2_CPM < 4 were excluded from all analyses. Variably expressed genes with log_2_CPM standard deviation ≥ 0.35 after adjustment for age and sex (via limma [34]), were included in the network analysis performed using MEGENA on the live samples. Recommended default software parameters were used, i.e., minimum module size set to 10 and using Pearson correlation for calculation of correlation in gene co-expression in building Planar Filtered Networks (PFN) [35]. MEGENA multiscale clustering analysis was performed in the manner of hierarchical division to dissect the PFNs into coherent modules with nested clusters at various scales of resolution. Each module was then tested for association with presence of SI (SSI ≥ 5 vs. SSI = 0) and separately for ideation severity within the ideator group. Note, the sample size for the low ideation group (i.e., SSI range 1-to-4, *N* = 6, Table 1) was limited and thus excluded from the comparison together with 2 MDD/NA subjects who had no SSI score, yet still included in the MEGENA analysis.

For each module, gene expression patterns were represented by an eigengene, i.e., the first principal component of the expression levels computed using prcomp function in R. Differences in eigengene expression between the group with moderate to elevated SI with SSI ≥ 5 (denoted as the high-SI group, *n* = 46) vs. the non-ideator group (SSI = 0, *n* = 46) was tested using the Wilcoxon rank sum test. Within the high-SI group, Spearman correlation coefficient with the eigengene expression was computed to assess the association of suicidal ideation severity with eigengene expression for each module. For modules found to be significantly associated with severity of SI, post-hoc analyses were also performed to test if observed associations with suicidal ideation severity persisted after adjusting for participants’ depression severity, measured by BDI and HAM17 with suicide-related items within these instruments removed. Analyses were likewise adjusted for participants‘ age of onset of major depression, number of episodes of major depression, subtypes of major depression (melancholic, or atypical), sleep quality (measured as BDI item 16), tiredness (measured as BDI item 17), or RIN. These analyses were run in separate models using robust regression via lmrob function from robustbase [36] in R, with the eigengene as the response, ideation severity as the predictor, and measurements mentioned above as the covariate. Moreover, post-hoc analyses adjusting for smoking status and medication use were not performed. Since most living subjects in the present study were nonsmokers (*n* = 90, 90%), smoking status and degree were not considered in post-hoc analyses. Additionally, since participants on medication (*n* = 22) consisted of a high proportion of the high-risk subgroup, excluding them in a post-hoc sensitivity analysis was not practical and thus not considered. Further, in light of the complex, multidimensional medication information (with many low-frequency cells for combinations of medications) covariate adjustment for individual medications or even families of medications was also not practical. For the postmortem brain RNA-seq data, analyses were performed on the same list of genes used in the live sample MEGENA analysis. Specifically, age and sex adjusted log_2_CPM gene expression data were used to compute the eigengene using eigenvector coefficients obtained from MEGENA network analysis from the live samples. For comparison of modules’ eigengene expression between suicide decedent and non-psychiatric non-suicide cases, robust Cohen’s d (via d.robust from pysch [37]) effect sizes were computed and reported, instead of performing significance testing, due to sample size limitations. In a secondary analysis, the effect sizes of group comparison (suicide decedent vs. non-suicide control) were computed as the standardized coefficient in a robust regression model with the adjustment of sample RIN, PMI, and pH.

### Ingenuity pathway and upstream regulator analysis

All unnested modules significantly associated with suicidal ideation and severity (*p* < 0.05) were included in Ingenuity Pathway Analysis (IPA, QIAGEN Inc.,) [38], using following thresholds: pathways that were significantly (Benjamini Hochberg adjusted BH *p* < 0.1) upregulated (z-score > 2) or downregulated (z-score < −2). Results of clusters with significant pathway were reported as circos plots using GOChord function from GOplot [39] package in R. For upstream analysis, the following thresholds and filtering were used: *p* < 0.05, |z-score | > 2 and upstream regulators were filtered for genes, RNAs, and proteins. All *p*-values for IPA were computed using Fisher’s exact tests.

## Results

**Fig. 1:**
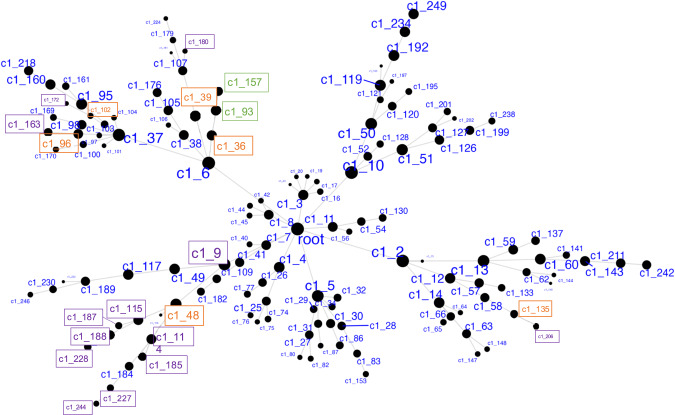
Hierarchical network structure of co-expressed gene clusters identified via MEGENA. Highlighted in orange are the 6 unnested parent clusters. Highlighted in purple are clusters showing significant differences in expression between High-SI and No-SI. In green are 2 nested clusters showing significant association with suicidal ideation severity among High SI.

**Fig. 2:**
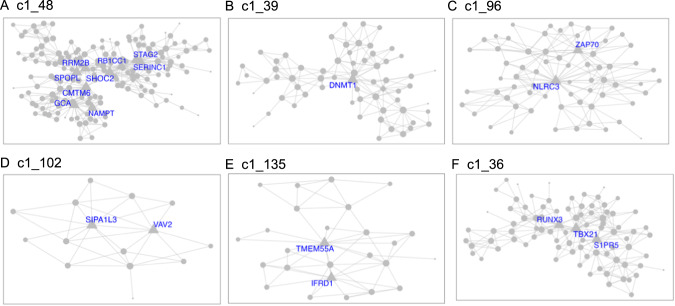
Unnested parent gene clusters associated with suicide. MEGENA modules **A**–**E** identified from suicide ideation group comparisons between high-SI vs. no-SI groups. Module **F** corresponds to ideation severity within the high-SI group. Hub genes are denoted with corresponding gene symbols.

**Fig. 3:**
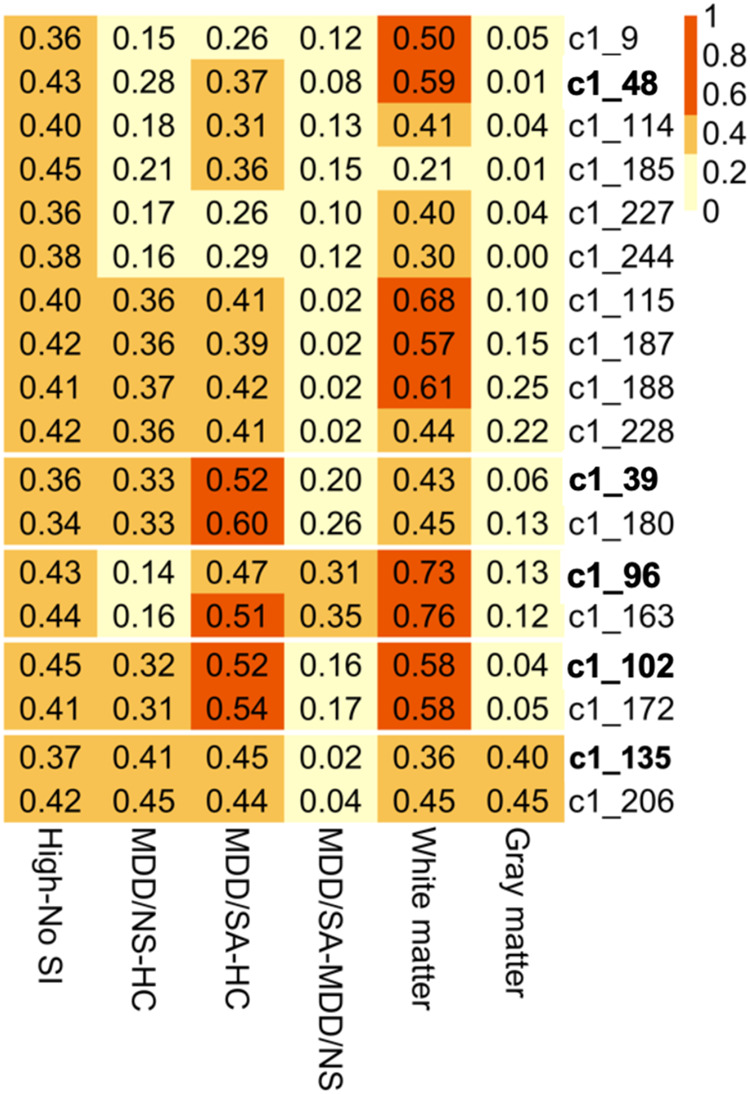
Heatmap of effect sizes from group comparisons. Effect sizes shown as absolute values of Cohen’s d from group comparisons of clusters’ eigengene expression, with column labels depicting group comparisons (last two columns show white/gray matter data contrasting samples of suicide death vs. non-psychiatric non-suicide death).

Demographics and relevant clinical measurements for the *N* = 100 live subjects are shown in Table 1, by recruitment group. Groups did not differ by age, sex, race, ethnicity, smoking status, and RNA quality, but as expected, differed on depression and suicidal ideation scales. Of these, 92 were included in gene network MEGENA analyses: 46 participants who reported elevated suicidal ideation (denoted as high-SI with SSI ≥ 5), and 46 who endorsed no ideation (denoted as no-SI, SSI = 0), irrespective of diagnostic status. To identify gene expression signatures associated with SI, peripheral blood gene co-expression analysis was performed in live samples. Using 2740 variably expressed genes from the live samples, the MEGENA analysis produced 135 modules ranging from 10 to 607 genes per module (Fig. 1). Eigengenes representing the overall gene expression pattern of each module were computed and compared between the high-SI vs. the no-SI groups using the Wilcoxon rank sum test, resulting in 18-modules associated with SI (Table S3). Of these, 5 modules with no overlapping genes (denoted as “unnested-modules”) were identified (c1_48, c1_39, c1_96, c1_102 and c1_135, *p* < 0.05, see Fig. 2A–E and Table S3). The parent c1_9 module consisting of 278 genes associated with SI contained two child clusters (i.e, c1_48 and c1_49 modules) in which the 113 gene c1_49 module showed no association with SI (*p* = 0.1586); MEGENA identified the SI-associated module c1_48 with superior specificity, which was then used for downstream functional analyses. To determine whether these modules’ associations with SI could be explained by diagnostic group or depression severity-related differences, post-hoc pairwise analyses were performed by comparing each module’s eigengene across the three groups (HC vs. MDD/NS vs. MDD/SA) using the Kruskal Wallis test. Two modules with significant group differences (c1_39, *p* = 0.0493; and its child cluster c1_180, *p* = 0.0458) showed significant medium sized difference between MDD/SA vs. HC (*p* = 0.0421, d = 0.52, Table S3 and Fig. 3) for c1_39 and trend-wise medium-sized difference between MDD/SA vs. HC (*p* = 0.0556, *d* = 0.60, Table S3 and Fig. 3) for c1_180. No significant differences were observed for the remaining two comparisons for the modules c1_39 and c1_180 (*p* > 0.05, Table S3).

Amongst the high-SI participants, 3 nested modules (Table S4) were associated with ideation severity, corresponding to modules c1_36 (r_s_ = −0.32, *p* = 0.0310, Fig. 2F) and corresponding child modules c1_93 (r_s_ = −0.32, *p* = 0.0278), and c1_157 (r_s_= 0.34, *p* = 0.0205). The associations between ideation severity and gene expression patterns for these three modules remained significant after adjusting for depression severity, sleep quality, tiredness, subtypes of major depression, and RIN (Table S4). Ideation severity in high-SI groups was significantly associated both with participants‘ age of onset of major depression (r_sp_ = −0.53, *p* = 0.0004), and number of episodes of major depression (r_sp_=0.36,*p* = 0.0202), which also showed moderate association with eigengene expression for these three modules (Table S4). However, including ideation severity together with age of onset of major depression (c1_36: SSI *p* = 0.3648, age of onset *p* = 0.2704; c1_93: SSI *p* = 0.3560, age of onset *p* = 0.2765; c1_157: SSI *p* = 0.3615, age of onset *p* = 0.2571, Table S4) or number of episodes in the model (c1_36: SSI *p* = 0.1437, number of episodes *p* = 0.8010; c1_93: SSI *p* = 0.1319, number of episodes *p* = 0.8463; c1_157: SSI *p* = 0.1309, number of episodes *p* = 0.8665, Table S4), with the eigengene expression as the response variable for the three modules separately, none of these factors remained significant.

Gene co-expression modules associated with high SI in peripheral blood were evaluated in both gray and white matter tissue from suicide decedents and non-psychiatric non-suicide controls using whole genome transcriptome data. The gray matter samples consisted of 21 suicide cases (8 female, 38%) and 29 non-suicide controls (6 female, 21%) with average ages of 52.05 (sd = 21.74) and 43.52 (sd = 21.26) respectively. Additionally, white matter samples comprised 15 cases (5 female, 33%) and 9 controls (3 female, 33%) with average ages of 53.80 (sd = 14.64) and 53.78 (sd = 11.88) respectively. It should be noted that for the postmortem cases, no group differences were observed for age (*p* = 0.9286), sex (perfectly balanced, *p* = 1.0), PMI (Wilcoxon *p* = 0.7649), pH (Wilcoxon *p* = 0.9523) or RIN (*p* = 0.7916) for the white matter, and similarly gray matter tissues (age: *p* = 0.1741, sex: *p* = 0.3012, PMI: *p* = 0.1182, pH: Wilcoxon *p* = 0.3255, RIN: Wilcoxon *p* = 0.3515). Due to the limited sample size of the postmortem cases, rather than testing for significance, high SI-associated modules from blood were evaluated using effect sizes, namely the robust Cohen’s d statistic. In white matter cases, medium effect sizes were detected for 9 high-SI associated modules (Fig. 3, Table S3). Specifically, amongst the five unnested modules in white matter tissue, the effect size ranged from a minimum of 0.36 for c1_135 to a maximum effect size of 0.73 for c1_96 (Fig. 3). In gray matter tissue, effect sizes ranging from negligible (*d* = 0.01 for c1_48) to small (*d* = 0.4 for c1_135) were observed for the unnested modules (Fig. 3, Table S3). The effect sizes from the secondary analyses where we adjusted for sample PMI, pH, and RIN were comparable to the robust Cohen’s d mentioned above (Table S3).

**Fig. 4:**
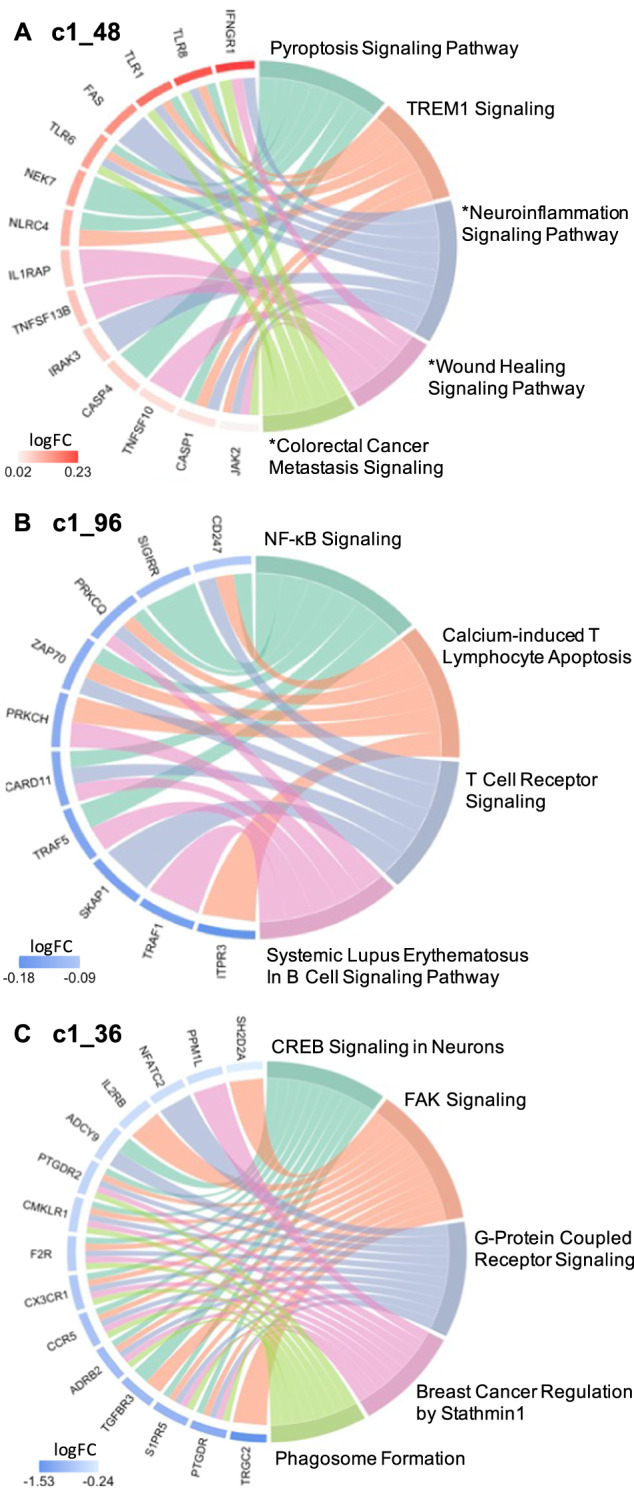
Circos plots show relationships between genes (left side) and significant IPA canonical pathways (right side). Significant IPA canonical pathways are identified for 2 SI group associated clusters (Panel A, B) and 1 suicide severity associated cluster (Panel C), based on |*Z* | > 2 and BH *p* < 0.1. Pathways marked with an asterisk (*) indicate an uncorrected *p* < 0.05 but BH *p* > 0.1. log FC = log 2 of fold change expression.

Gene ontology analyses were performed to delineate the functional importance of the genes within the modules associated with high SI and SI severity, using a significant threshold Benjamini Hochberg adjusted BH *p* < 0.1 chosen a priori. Pathway analysis, performed via IPA using genes from the unnested modules (Fig. 2), showed consistent gene expression differences in both blood and brain (in white matter only). Notably, the high SI and high suicide-related modules c1_48 and c1_96 showed enrichment of genes involved in immune and inflammatory pathways. Inflammatory pathways, including pyroptosis signaling (BH *p* = 0.0015, *Z* = 2.65), TREM1 signaling (BH p = 0.0016, *Z* = 2.45), and neuroinflammation pathways (trend-wise significant, BH *p* = 0.125, *Z* = 2.83), were all upregulated in the high SI-associated module c1_48 (Fig. 4A, Table S5). Additionally, the high SI-and-suicide associated module c1_96 showed enrichment of genes in the NF-κB (BH *p* = 0.0593, *Z* = −2.24), T cell receptor (BH *p* = 0.0593, *Z* = −2.24), and B cell signaling immune pathways (BH *p* = 0.0678, *Z* = −2.24) (Fig. 4B, Table S5). Module c1_36, associated with SI severity showed enrichment of genes involved in cell cycle regulation i.e., CREB signaling (BH *p* < 0.0001, *Z* = −3.61), FAK signaling (BH *p* < 0.0001, *Z* = −3.87) and Stathmin regulation (BH *p* = 0.0001, *Z* = −3.46) (Fig. 4C, Table S5). No significant findings were detected in pathway analyses of genes within the c1_39 (associated with suicide attempt), and c1_102 and c1_135 modules.

## Discussion

In the present study, we investigated the associations between coordinated gene expression clusters and suicide ideation using whole genome transcriptional data from both live and postmortem samples. Given the labile nature of gene expression and SI dynamics, we chose to focus on identifying transcript profiles that are associated with suicidal ideation presence and severity. In addition to transcriptome data from peripheral blood from live participants, whole genome transcriptome data from gray and white matter postmortem brains of suicide decedents and controls were used for cross-comparison of ideation with suicide death. Blood gene co-expression network analysis revealed a total of 18 SI-associated modules transdiagnostically, of which 9 were detected with moderate effect sizes in white matter, while none were in gray matter.

Across the SI-associated modules, gene ontology analyses demonstrated enrichment of genes involved in immune and inflammatory processes. Our findings indicate that in peripheral blood, ideators show enhanced inflammatory signatures of molecules related not only to adaptive but also to innate immune responses, such as recognition of pathogen or damage-associated molecular patterns (PAMPs/DAMPs) and activation of proinflammatory signals. A salient finding in the present study is the identification of peripheral blood monocyte gene co-expression related to inflammatory processes in high-SI participants, with enrichment of genes in Pyroptosis Signaling, TREM1 Signaling, NF-κB signaling, T Cell Receptor Signaling, Systemic Lupus Erythematosus In B Cell Signaling, Calcium-induced T Lymphocyte Apoptosis, and trend wise enrichment of genes in neuroinflammation pathways in the high SI vs. no SI groups. This is reflected in modules c1_48 and c1_96, with a number of genes overlapping across these pathways shown in Fig. 4A, B. Notably in the upregulated pathways from module c1_48 (Fig. 4A), the Toll-like receptor (*TLR*) genes shared across these pathways in the high-SI compared to the no-SI group is consistent with prior findings, in which mRNA and protein expression of *TLR* loci were higher in serum and prefrontal cortex of depressed suicide decedents compared with non-suicide decedents, respectively [40, 41]. Innate immune receptors such as *TLR*s participate in the initiation of the immune activation cascade, leading to the production of cytokines in the brain. Growing evidence supports the role of these receptors in facilitating the brain to mount immune responses during systemic infection and neuronal injury [42] as well as in mood disorders [43].

Furthermore, of the 16 hub genes (highly interconnected, Table 2) identified within these SI-associated modules, 13 are novel in terms of association with suicidal ideation. Notably, the gene *DNMT1*, a hub gene in cluster c1_39, encodes the protein for DNA-methyltransferase 1 (*DNMT1*). Decreased *DNMT1* expression has been reported in the brain of suicide decedents [44] with altered expression also detected in the limbic system and brainstem of suicide decedents as compared with controls. Although none of the individual genes identified in this study showed robust fold changes in terms of the magnitude of gene expression differences between the high-SI vs. no-SI groups, they do show coordinated gene expression differences associated with suicidal ideation, in pathways previously implicated in neurodegenerative and psychiatric disorders evidenced by both animal models and human studies [45–56]. Thus, they can be targeted for future studies including for consideration as potential predictors or pharmacological targets in preclinical and clinical suicide research studies.

**Table 2:** Hub genes of clusters with differential expression pattern between high-SI and no-SI group.

Cluster	Hub genes (degree of intra-module connectivity)	Role of hub genes
C1_39	DNMT1 (17)	Gene expression regulation (DNA methylation)
C1_48	SHOC2 (30), CMTM6 (23), RRM2B (22), SPOPL (21), GCA (20), STAG2 (19), SERINC1 (18), RB1CC1 (16), NAMPT (16)	Metabolism of lipid, protein, DNA and nicotinamide adenine dinucleotide (NAD); Innate immunity regulation; Cell cycle regulation; DNA repair
C1_96	NLRC3 (33), ZAP70 (21)	Immunity regulation (both innate and adaptive responses): cytosolic regulator of innate immunity; Motility, adhesion and cytokine expression of mature T-cells
C1_102	SIPA1L3 (11), VAV2 (9)	Cell proliferation; Endothelial cell migration Angiogenesis
C1_135	TMEM55A (15), IRFD1 (11)	Metabolism of lipids; Cell proliferation

Across the 18 SI-associated modules found in peripheral blood, 9 modules were also altered in white matter with moderate effect sizes, specifically in ventral PFC white matter *postmortem* in suicide decedents. The gene ontology analyses demonstrated enrichment of genes involved in both innate and adaptive immune responses shown in modules c1_48 and c1_96 as discussed above. Since normal white matter is essential for the brain’s executive function, a greater focus on white matter integrity appears indicated for suicide research. Intact white matter is critical for functioning of prefrontal cortical areas related to attention, self-control, planning, decision-making, mood regulation, and other higher cognitive abilities [57]. Loss of myelin that affects disruption of the connectivity between the PFC and the limbic system, can lead to perceptual misrepresentations, misjudgment, impulsivity, and a distorted cognitive appraisal of reality. In vivo studies of the frontal lobe in depression and suicidality report reduced metabolic response [58, 59] and disrupted brain connectivity in gray and white matter of those who attempt or die by suicide [60]. Early studies using structural magnetic resonance imaging (SMRI) also support white matter alterations in patients with a history of suicide attempt by showing an increase in white matter hyperintensities [61–63]. Diffusion tensor imaging (DTI) studies have shown diminished structural integrity of white matter that provides fronto-limbic connections in suicide attempters. Decreased fractional anisotropy (FA) has been reported in ventral frontal white matter in suicide attempters, including within the region of the uncinate fasciculus that carries major ventral PFC-amygdala connections, with associations with impulsivity [64, 65]. Our findings of gene modules related to suicidal ideation detected in white matter of suicide decedents may also relate to suicidal behavior, as 41% of participants with suicidal ideation in the present study had a prior history of suicide attempt, as compared to 9% in the no ideation group.

White matter cells of the human CNS are comprised mostly of glia, including oligodendrocytes, astrocytes, and microglia that participate in the mounting of inflammatory responses and processes in the CNS [66]. As the most abundant cells in the CNS, glial cells interact with neurons and immune cells, as well as brain microvascular endothelial cells. Glial cells, notably microglia, function as resident innate immune cells, wherein white matter of suicide postmortem cases increased densities of activated microglia [67] and increased microglial priming and macrophage recruitment [68] have been reported. Microglial activation can be stimulated through the transmission of inflammatory signals from the periphery to the brain via sensory afferent projections or humoral transmigration through a potentially impaired blood-brain barrier [69, 70], where abnormalities in the blood-brain barrier and endothelial cells have also been implicated in suicide [33, 71]. In an activated state, microglia adopt different phenotypes and, in response to PAMPs and DAMPs [72] that bind to *TLR* and activate *NFkB*, secrete numerous pro-inflammatory cytokines and chemokines [73]. Also affected by PAMPs and DAMPs, brain microvascular endothelial cells constituting the blood-brain barrier participate in inflammatory processes and cytokine secretion through altered activity of *TLR*s [74]. Such inflammatory processes can be induced by CNS injury, autoimmune disorders, or infectious agents that are associated with increased risk of suicide [75–79]. In particular, infections can affect the brain directly (e.g., via influenza B virus14 and the parasite Toxoplasma gondii) [80–83] or from the periphery by generating molecular mediators of inflammation that cross from the periphery into the brain [84] and thereby increase the risk of suicide. Cumulative evidence from population-based studies shows that infection and TBI are predictive of suicide risk and death by suicide [75, 78, 79, 85]. The dynamic cross-talk between peripheral inflammation and endothelial and microglial cells via potentially altered TLR activity induced neuroinflammation is consistent with our findings of increased expression of inflammatory genes both in blood and in white matter, as observed in modules c1_48 and c1_96. Additionally, findings related to altered mRNA expression of *TLR*s associated with elevated SI in the present study and in postmortem brains from suicide decedents [41] can further inform the understanding of upstream mechanisms in neuroinflammation that lead to abnormalities in cytokine expression in suicidal patients across the continuum of suicide risk.

This study also identified gene co-expression modules associated with suicidal ideation severity (i.e., 3 nested modules corresponding to modules c1_36, c1_93, and c1_157). Remarkably, amongst the high-risk SI participants, these modules were associated with age of onset of major depression and number of depressive episodes. These findings support the idea that such patients are likely to be a more severe subtype. Indeed, this is in line with the growing body of evidence supporting that early onset depression is heritable, showing genetic risk loci associated with early onset depression contributing to increased risk for psychiatric disorders [86]. Family history of depression is associated with early onset of the disorder, in that early onset parental depression as compared to late onset parental depression confers a greater risk for depression in offspring [87]. Importantly, early onset depression is correlated with greater depressive severity and chronicity, as well as increased suicidality [88, 89]. Functional analyses of these ideation severity co-expressed modules have identified biologically meaningful genetic pathways that show coordinated expression specifically in the c1_36 module with enrichment of genes involved in cell cycle regulation including *CREB* signaling, FAK signaling, and Stathmin regulation (Fig. 4C and Table S5). Notably, altered cell cycle regulation previously has been linked to suicidal ideation and behavior in both peripheral blood and postmortem brain studies [90–92]. One of the early seminal findings that resulted from genome-scale transcriptional regulatory studies in peripheral blood identified the gene *SKA2* (Spindle and Kinetochore Associated Complex Subunit 2) as a potential biomarker of suicide risk and ideation [93] that prospectively predicted suicidal ideation and behavior in psychiatric patients [94]. Expression of *SKA2* is regulated by transcription factors including *CREB*, a nuclear transcription factor that also regulates transcription activity of neuronal survival and expression of different growth factors [95]. *CREB* expression is also downregulated in multiple major psychiatric disorders, including bipolar disorder, schizophrenia, and major depressive disorder [96–98], and decreased protein and mRNA expression of *CREB* is observed in postmortem brain of depressed suicide decedents [98]. These findings are consistent with the observed downregulation of the *CREB* signaling pathway associated with SI severity in the present study.

Several strengths of the study design add confidence to the findings. The design allows comparison of genomic biology in peripheral blood related to suicidal ideation in brain to suicide death. The ideation severity findings were not attributable to depression severity. This study also has several limitations. The samples (although well-characterized through psychological assessments for live participants and psychological autopsy for postmortem cases), were not characterized for history or presence of infections, autoimmune disorders, or CNS injuries that can potentially impact SI-associated gene co-expression modules identified in the present study. As is often the case in studies of suicidality, comorbid medical and mental health conditions, as well as medications to mitigate risk in these conditions, are also a potential confound both in study participants with SI and in the postmortem brain cases of suicide decedents and non-suicide controls. While varied comorbid conditions, medications, and life experiences across different studies may limit potential reproducibility, these findings are likely generalizable as the study participants were representative of the population at-risk. Also, as is often the case in human postmortem brain studies sample sizes are small, and the measure of RNA quality RIN (RNA integrity number) can be highly variable. In this study, the RIN values ranged from 2.9 to 9.0 for gray matter and 4.0–9.1 for white matter, with all RNAseq data passing quality control, indicating that RNA of low RIN can result in reliable RNA-seq data. This finding aligns with previous reports showing that RIN was not a sensitive measure of RNA quality for postmortem human brains [99]. RIN is not a sensitive measure of RNA quality for substantially degraded samples because, first, the RIN score relies heavily on the amount of 18 S and 28 S ribosome RNAs but fails to measure the mRNA integrity directly, and second, the RIN is an overall assessment of RNA quality, and cannot serve as a specific criterion to adjust for differential RNA degradation among transcripts in downstream gene expression analyses. This limits its application in both pre-sequencing RNA sample screening and post-sequencing RNA-seq data analysis. Lastly, across the SI-associated modules, individual genes within each module did not show significant expression differences in the SI vs. no-SI groups. This is not unexpected, since gene network approaches by design identify group(s) of genes that are changing in the same direction and magnitude, even if these changes are small. Modules of co-expressed genes thus identified in the present study will likely be co-regulated or may belong to the same functional pathway, which needs to be validated in future studies with additional experiments in vivo using animal models or in vitro using cell lines to confirm their effects on genes within relevant modules.

In conclusion, findings from this study suggest that gene expression networks are a valuable tool for identification of common pathways between suicide, psychiatric disease, inflammatory and autoimmune conditions, as well as factors unique to suicidality in the continuum of risk from suicidal ideation, suicidal behavior, and suicide. The high SI-associated co-expressed gene modules implicated with immune processes suggest potentially significant signals driving biological and molecular changes both in the periphery and in the CNS (especially in the white matter), which can potentially distinguish patients with elevated SI that may be at imminent risk for suicide. Identification of such gene expression networks occurring in both peripheral and CNS tissue as demonstrated in the present study can be integrated with other clinical measures in translational and biomarker studies of SI to improve models for suicide risk prediction.

### Supplementary information

Supplemental Tables and Figures Legends

Supplemental Table 1

Supplemental Table 2

Supplemental Table 3

Supplemental Table 4

Supplemental Table 5

Supplemental Figure 1

Supplemental Figure 2

## Data Availability

Codes used for statistical analyses are made available upon request.

## Data availability

The data that supports the findings of this study is uploaded to GEO (GSE247998 for live sample; GSE248260 for the white matter sample). Please us the token “afuhuwysxfcptqr” for GSE247998 and the token “grqhyeeodpcfnav” for GSE248260 to gain reviewer access to the data.
