# Supplementary figures and images for: Brain and blood transcriptome profiles delineate common genetic pathways across suicidal ideation and suicide
Source: Mol Psychiatry. Author manuscript; Available in PMC 2024 Jun 21. (PMC11189724; doi:10.1038/s41380-024-02420-z)

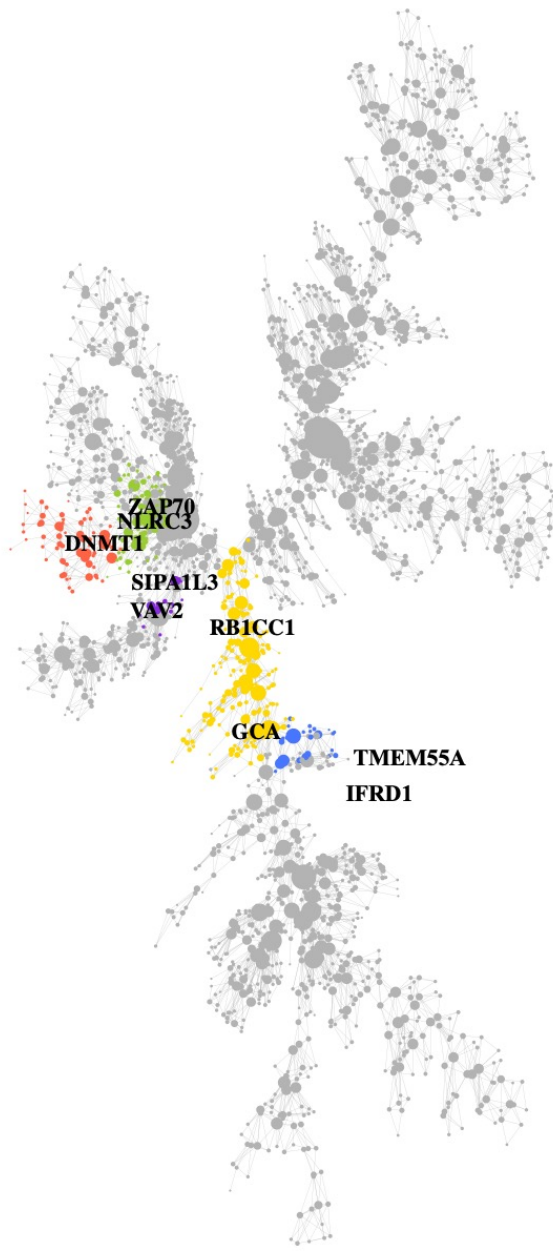

- c1\_39
- c1\_48
- c1\_96
- c1\_102
- c1\_135

Supplement: Supplementary Figure 1 [file NIHMS1995941-supplement-Supplementary_Figure_1.pdf]

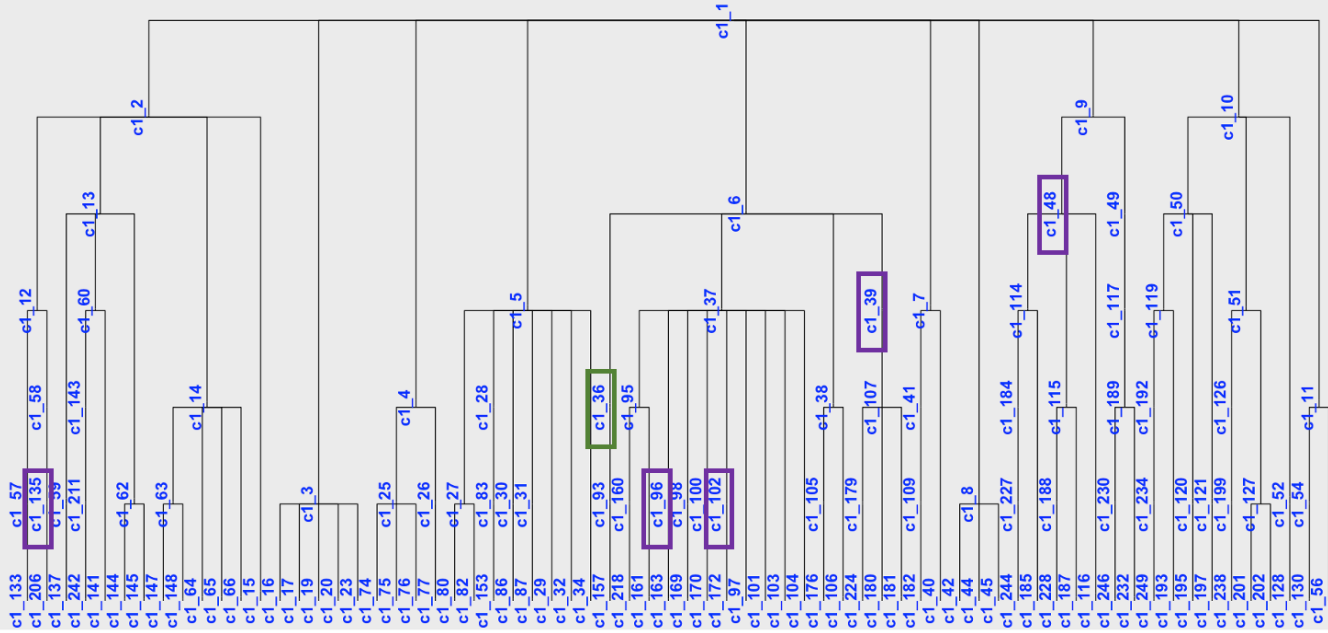

Supplement: Supplementary Figure 2 [file NIHMS1995941-supplement-Supplementary_Figure_2.pdf]
