# Supplementary material for: Brain and blood transcriptome profiles delineate common genetic pathways across suicidal ideation and suicide
Source: Mol Psychiatry. 2024 Jan 26;29(5):1417–26. doi: 10.1038/s41380-024-02420-z (PMC11189724; doi:10.1038/s41380-024-02420-z)
Supplement: Supplementary file 1 [file 41380_2024_2420_MOESM1_ESM.docx]

**Legends: Supplementary Tables** 

**Table S1:** Live participants prior and current medication history within 14 days of the blood draw.

**Table S2:** Postmortem white matter cases providing information on group/suicide status, cause of death, and brain toxicology, PH, PMI (postmortem interval), etc.

**Table S3:** 18 modules identified from high- vs no- SI group comparison.  The largest module, c1_9, had two direct child modules, with only one (c1_48, and its corresponding child clusters) differing significantly by high- vs. no-SI groups.  When performing functional gene ontology analyses on the 5 un-nested clusters, the child module c1_48 was used rather than c1_9 for greater specificity.  

**Table S4:** 3 nested modules identified from ideation severity analysis within High-SI group.

**Table S5:** Shows significant results from IPA canonical pathway and upstream regulator analyses of the 5 un-nested clusters associated with high-SI as well as 1 cluster associated with suicidal ideation severity. 

**Legends: Supplementary Figures**

**Figure S1:** MEGENA network with five clusters from ideation group analysis highlighted and examples of hub genes labeled.

**Figure S2:** Hierarchical MEGENA Network Structure. Highlighted in purple are 5 parent clusters showing significant difference in expression between High-SI and No-SI. In green is the parent cluster showing significant association with suicidal ideation severity among High SI.
